# 9-Eth­oxy-1,5,13-trimethyl-8,10-dioxa­tetra­cyclo­[7.7.1.0^2,7^.0^11,16^]hepta­deca-2,4,6,11,13,15-hexa­ene

**DOI:** 10.1107/S1600536809032747

**Published:** 2009-08-22

**Authors:** Ewa Maria Nowakowska-Bogdan, Ewa Wicher, Krzysztof Ejsmont, Jacek Zaleski

**Affiliations:** a Institute of Heavy Organic Synthesis "Blachownia", Energetyków 9, 47-225 Kędzierzyn–Koźle, Poland; b Faculty of Chemistry, University of Opole, Oleska 48, 45-052 Opole, Poland

## Abstract

The reaction of ethyl acetoacetate with *meta*-cresol in an acidic ionic liquid yielded a complex mixture of condensation products. 4,7-Dimethyl­coumarin and the title compound, C_20_H_22_O_3_, were isolated. The title compound shows chemical but not crystallographic mirror symmetry. The two aromatic rings are inclined at an angle of 73.55 (6)°.

## Related literature

For related structures, see: Klei *et al.* (1995 ▶); Vijayalakshmi *et al.* (2001 ▶).
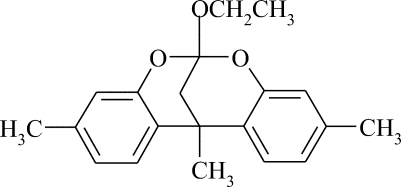


## Experimental

### 

#### Crystal data

C_20_H_22_O_3_
*M* *_r_* = 310.38
Monoclinic, 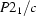

*a* = 14.3718 (6) Å
*b* = 11.6446 (5) Å
*c* = 10.2260 (4) Å
β = 96.901 (4)°
*V* = 1698.96 (12) Å^3^
*Z* = 4
Mo *K*α radiation
μ = 0.08 mm^−1^
*T* = 90 K
0.25 × 0.20 × 0.10 mm

#### Data collection

Oxford Diffraction Xcalibur diffractometer
Absorption correction: none
9989 measured reflections
2985 independent reflections
1751 reflections with *I* > 2σ(*I*)
*R*_int_ = 0.031

#### Refinement

*R*[*F* ^2^ > 2σ(*F* ^2^)] = 0.047
*wR*(*F* ^2^) = 0.126
*S* = 0.95
2985 reflections
252 parameters
H atoms treated by a mixture of independent and constrained refinement
Δρ_max_ = 0.27 e Å^−3^
Δρ_min_ = −0.23 e Å^−3^

### 

Data collection: *CrysAlis CCD* (Oxford Diffraction, 2008 ▶); cell refinement: *CrysAlis RED* (Oxford Diffraction, 2008 ▶); data reduction: *CrysAlis RED*; program(s) used to solve structure: *SHELXS97* (Sheldrick, 2008 ▶); program(s) used to refine structure: *SHELXL97* (Sheldrick, 2008 ▶); molecular graphics: *SHELXTL* (Sheldrick, 2008 ▶); software used to prepare material for publication: *SHELXL97*.

## Supplementary Material

Crystal structure: contains datablocks global, I. DOI: 10.1107/S1600536809032747/bt2970sup1.cif

Structure factors: contains datablocks I. DOI: 10.1107/S1600536809032747/bt2970Isup2.hkl

Additional supplementary materials:  crystallographic information; 3D view; checkCIF report

## Figures and Tables

**Fig. 1.**
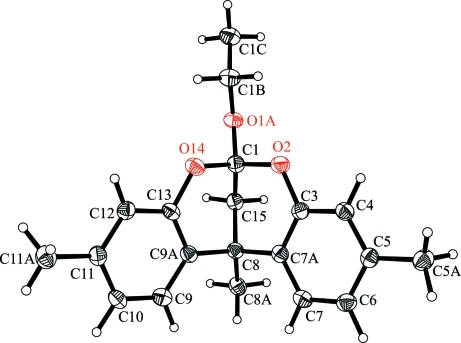
The molecular structure of the title compound showing 50% displacement ellipsoids (arbitrary spheres for the H atoms).

## supplementary crystallographic information

### Experimental

Anhydrous aluminium chloride (16.0 g, 60 mmol of Al_2_Cl_6_) and 1-*n*-butyl-3-methyl-imidazolium chloride (10.5 g, 60 mmol of [bmim]Cl) were mixed under dry nitrogen atmosphere. Ionic liquid ([bmim] Al_2_Cl_7_) was formed in the exothermic reaction of two solid substrates. The melt of *meta*-cresol (6.3 ml, 60 mmol) and ethyl acetoacetate (7.5 ml, 60 mmol) was dissolved in the ionic liquid and maintained at ambient temperature for 5 days. A yellow, viscous liquid was poured on ice and an opaque solution was extracted twice with methylene chloride. The organic solution was extracted with diluted sulfuric acid (25 ml of 3*M* H_2_SO_4_) and water to remove aluminium compounds. It was dried over anhydrous magnesium sulfate and adsorbed on silica gel (Kieselgel H, Fluka). The crude reaction mixture was chromatographed on the short column (5.5 by 15 cm) using benzene as the eluent. The first fraction, after evaporation and crystallization from n-hexane, gave title compound (I) (1.11 g, 12%) as colourless prisms, m.p. 146–153°C. Recrystallization from isooctane raised m.p. to 153–155°C, the crystals were suitable for X-ray diffraction studies. MS, m/*z* (int.): 310 (38, *M*^+^), 295 (100), 281 (5), 267 (78), 264 (5), 249 (7), 239 (8), 223 (11), 203 (27), 175 (26). FTIR (KBr): 3037 (aromatic protons); 2985, 2969, 2937, 2909 (aliphatic C–H stretching vibrations); 1623, 1580, 1506 (benzene ring stretching); 1271, 1157, 1127, 1090, 1050, 1008 (C–O–C stretching vibrations); 886, 814 (out of plane hydrogen wagging in aromatic rings). ^1^H-NMR (DMSO-d6): 7.26, d 3 J = 7.4 Hz, 2H and 6.70, d ^3^J = 7.4 Hz, 2H (vicinal aromatic protons); 6.63, s, 2H (isolated aromatic protons); 4.04, q, ^3^J = 6.7 Hz, 2H and 1.26, t, 3 J = 6.7 Hz, 3H (*O*-ethyl group); 2.21, s, 2H (methylene bridge); 2.16, s, 6H (methyl groups bound to aromatic rings); 1.76, s, 3H (methyl group). ^13^C-NMR (CDCl_3_): 151.8 (C4, C16); 137.8 (C6, C14); 127.5 (C9, C11); 123.8 (C8, C12); 122.6 (C7, C13); 117.1 (C5, C15); 112.1 (C2); 58.5 and 15.7 (*O*-ethyl group); 38.8 (methylene bridge); 34.8 (C10); 21.1 (methyl groups on aromatic rings). The next fraction provided 4,7-dimethyl-coumarin as white crystals (1.32 g, 12.6%); m.p. 135–136°C (n-hexane). From the last fraction small amounts of 1,2-dihydro-4,7-dimethyl-4-(4-hydroxy-2-methylphenyl)-coumarin (isomer 2) were isolated (m.p. 211–212°C).

### Refinement

Methyl H-atoms were positioned geometrically and refined using a riding model allowed to rotate but not to tip with *U*_iso_(H) = 1.5*U*_eq_(C). The remaining H atoms were freely refined.

### Crystal data

**Table:**

C_20_H_22_O_3_	*F*(000) = 664
*M**_r_* = 310.38	*D*_x_ = 1.213 Mg m^−^^3^
Monoclinic, *P*2_1_/*c*	Mo *K*α radiation, λ = 0.71073 Å
Hall symbol: -P 2ybc	Cell parameters from 2985 reflections
*a* = 14.3718 (6) Å	θ = 2.7–25.0°
*b* = 11.6446 (5) Å	µ = 0.08 mm^−^^1^
*c* = 10.2260 (4) Å	*T* = 90 K
β = 96.901 (4)°	Plate, colourless
*V* = 1698.96 (12) Å^3^	0.25 × 0.20 × 0.10 mm
*Z* = 4	

### Data collection

**Table:**

Oxford Diffraction Xcalibur diffractometer	1751 reflections with *I* > 2σ(*I*)
Radiation source: fine-focus sealed tube	*R*_int_ = 0.031
graphite	θ_max_ = 25.0°, θ_min_ = 2.7°
Detector resolution: 1024 x 1024 with blocks 2 x 2 pixels mm^-1^	*h* = −17→17
ω scans	*k* = −13→13
9989 measured reflections	*l* = −12→6
2985 independent reflections	

### Refinement

**Table:**

Refinement on *F*^2^	Primary atom site location: structure-invariant direct methods
Least-squares matrix: full	Secondary atom site location: difference Fourier map
*R*[*F*^2^ > 2σ(*F*^2^)] = 0.047	Hydrogen site location: inferred from neighbouring sites
*wR*(*F*^2^) = 0.126	H atoms treated by a mixture of independent and constrained refinement
*S* = 0.95	*w* = 1/[σ^2^(*F*_o_^2^) + (0.0723*P*)^2^] where *P* = (*F*_o_^2^ + 2*F*_c_^2^)/3
2985 reflections	(Δ/σ)_max_ < 0.001
252 parameters	Δρ_max_ = 0.27 e Å^−^^3^
0 restraints	Δρ_min_ = −0.23 e Å^−^^3^

### Special details

**Table:**

Geometry. All e.s.d.'s (except the e.s.d. in the dihedral angle between two l.s. planes) are estimated using the full covariance matrix. The cell e.s.d.'s are taken into account individually in the estimation of e.s.d.'s in distances, angles and torsion angles; correlations between e.s.d.'s in cell parameters are only used when they are defined by crystal symmetry. An approximate (isotropic) treatment of cell e.s.d.'s is used for estimating e.s.d.'s involving l.s. planes.
Refinement. Refinement of *F*^2^ against ALL reflections. The weighted *R*-factor *wR* and goodness of fit *S* are based on *F*^2^, conventional *R*-factors *R* are based on *F*, with *F* set to zero for negative *F*^2^. The threshold expression of *F*^2^ > σ(*F*^2^) is used only for calculating *R*-factors(gt) *etc*. and is not relevant to the choice of reflections for refinement. *R*-factors based on *F*^2^ are statistically about twice as large as those based on *F*, and *R*- factors based on ALL data will be even larger.

### Fractional atomic coordinates and isotropic or equivalent isotropic displacement parameters (Å^2^)

**Table:**

	*x*	*y*	*z*	*U*_iso_*/*U*_eq_	
C1	0.24961 (15)	0.47696 (17)	0.2890 (2)	0.0235 (5)	
O1A	0.20501 (9)	0.37104 (11)	0.26845 (13)	0.0263 (4)	
C1B	0.17179 (18)	0.3422 (2)	0.1307 (2)	0.0317 (6)	
C1C	0.13069 (17)	0.22326 (18)	0.1300 (2)	0.0379 (6)	
H1C1	0.1089	0.2012	0.0411	0.057*	
H1C2	0.0791	0.2226	0.1816	0.057*	
H1C3	0.1778	0.1701	0.1668	0.057*	
O2	0.33294 (10)	0.47258 (12)	0.22253 (13)	0.0288 (4)	
C3	0.40517 (14)	0.55031 (17)	0.26325 (19)	0.0226 (5)	
C4	0.48180 (15)	0.55002 (18)	0.1881 (2)	0.0236 (5)	
C5	0.55726 (14)	0.62572 (17)	0.21702 (19)	0.0236 (5)	
C5A	0.64239 (15)	0.62257 (19)	0.1394 (2)	0.0294 (5)	
H51	0.6221	0.6032	0.0492	0.044*	
H52	0.6862	0.5660	0.1771	0.044*	
H53	0.6720	0.6966	0.1433	0.044*	
C6	0.55384 (16)	0.70296 (18)	0.3227 (2)	0.0271 (5)	
C7	0.47795 (15)	0.70088 (18)	0.3991 (2)	0.0253 (5)	
C7A	0.40231 (14)	0.62393 (17)	0.37198 (18)	0.0222 (5)	
C8	0.31708 (14)	0.62109 (17)	0.45321 (19)	0.0218 (5)	
C8A	0.34643 (15)	0.64395 (18)	0.60163 (19)	0.0276 (5)	
H81	0.2923	0.6397	0.6480	0.041*	
H82	0.3738	0.7190	0.6129	0.041*	
H83	0.3914	0.5873	0.6360	0.041*	
C9A	0.24205 (14)	0.70553 (17)	0.38933 (19)	0.0226 (5)	
C9	0.23106 (15)	0.81749 (18)	0.4337 (2)	0.0256 (5)	
C10	0.16298 (15)	0.89076 (19)	0.3686 (2)	0.0256 (5)	
C11	0.10309 (14)	0.85430 (17)	0.2566 (2)	0.0244 (5)	
C11A	0.03150 (16)	0.93435 (19)	0.1825 (2)	0.0341 (6)	
H111	−0.0282	0.8965	0.1681	0.051*	
H112	0.0515	0.9543	0.0992	0.051*	
H113	0.0260	1.0027	0.2334	0.051*	
C12	0.11309 (15)	0.74147 (18)	0.2120 (2)	0.0240 (5)	
C13	0.18201 (14)	0.66987 (17)	0.27796 (19)	0.0225 (5)	
O14	0.18724 (10)	0.56098 (11)	0.22264 (13)	0.0272 (4)	
C15	0.27228 (16)	0.50062 (18)	0.4352 (2)	0.0240 (5)	
H1B1	0.2258 (16)	0.3455 (18)	0.076 (2)	0.043 (7)*	
H1B2	0.1230 (15)	0.4022 (18)	0.094 (2)	0.033 (6)*	
H4A	0.4837 (14)	0.4925 (18)	0.116 (2)	0.028 (6)*	
H6A	0.6069 (14)	0.7588 (18)	0.3474 (19)	0.025 (5)*	
H7A	0.4784 (14)	0.7539 (18)	0.472 (2)	0.027 (6)*	
H9A	0.2729 (14)	0.8461 (17)	0.515 (2)	0.028 (6)*	
H10A	0.1543 (14)	0.9711 (18)	0.403 (2)	0.031 (6)*	
H12A	0.0748 (17)	0.7141 (19)	0.133 (2)	0.044 (7)*	
H15A	0.3208 (15)	0.4429 (19)	0.477 (2)	0.035 (6)*	
H15B	0.2132 (16)	0.4963 (17)	0.480 (2)	0.032 (6)*	

### Atomic displacement parameters (Å^2^)

**Table:**

	*U*^11^	*U*^22^	*U*^33^	*U*^12^	*U*^13^	*U*^23^
C1	0.0254 (12)	0.0176 (11)	0.0280 (11)	−0.0014 (9)	0.0055 (9)	0.0029 (9)
O1A	0.0305 (9)	0.0197 (8)	0.0285 (8)	−0.0036 (7)	0.0028 (7)	−0.0012 (6)
C1B	0.0363 (14)	0.0266 (13)	0.0319 (12)	−0.0039 (11)	0.0023 (11)	−0.0039 (10)
C1C	0.0399 (15)	0.0277 (14)	0.0451 (15)	0.0006 (11)	0.0005 (12)	−0.0074 (11)
O2	0.0273 (8)	0.0280 (9)	0.0324 (8)	−0.0055 (7)	0.0085 (7)	−0.0072 (7)
C3	0.0233 (12)	0.0188 (11)	0.0248 (11)	0.0009 (9)	−0.0014 (9)	0.0015 (9)
C4	0.0259 (12)	0.0221 (12)	0.0225 (11)	0.0016 (9)	0.0015 (9)	−0.0008 (9)
C5	0.0229 (11)	0.0227 (12)	0.0246 (11)	0.0031 (10)	0.0003 (9)	0.0041 (9)
C5A	0.0280 (12)	0.0323 (13)	0.0279 (12)	−0.0039 (10)	0.0031 (10)	−0.0008 (10)
C6	0.0273 (13)	0.0218 (12)	0.0311 (12)	−0.0017 (10)	−0.0004 (10)	0.0003 (10)
C7	0.0307 (13)	0.0207 (12)	0.0243 (11)	0.0014 (10)	0.0024 (10)	−0.0015 (9)
C7A	0.0244 (11)	0.0203 (11)	0.0214 (11)	0.0032 (9)	0.0014 (9)	0.0027 (9)
C8	0.0228 (11)	0.0195 (11)	0.0235 (11)	−0.0008 (9)	0.0045 (9)	0.0001 (9)
C8A	0.0309 (12)	0.0272 (13)	0.0254 (11)	0.0037 (10)	0.0068 (10)	0.0010 (10)
C9A	0.0243 (12)	0.0203 (11)	0.0237 (11)	−0.0001 (9)	0.0053 (9)	0.0002 (9)
C9	0.0306 (13)	0.0245 (12)	0.0224 (11)	−0.0017 (10)	0.0060 (10)	−0.0003 (10)
C10	0.0302 (12)	0.0192 (12)	0.0291 (12)	0.0022 (10)	0.0105 (10)	−0.0011 (10)
C11	0.0230 (11)	0.0218 (12)	0.0292 (11)	0.0002 (9)	0.0060 (9)	0.0038 (9)
C11A	0.0331 (13)	0.0281 (13)	0.0407 (13)	0.0029 (11)	0.0023 (11)	0.0058 (10)
C12	0.0215 (12)	0.0226 (12)	0.0281 (12)	−0.0022 (10)	0.0036 (10)	0.0030 (9)
C13	0.0276 (12)	0.0174 (11)	0.0239 (10)	−0.0001 (9)	0.0084 (9)	−0.0005 (9)
O14	0.0340 (9)	0.0214 (8)	0.0251 (8)	0.0031 (7)	−0.0007 (7)	−0.0010 (6)
C15	0.0247 (12)	0.0221 (12)	0.0258 (11)	0.0006 (10)	0.0054 (10)	0.0016 (9)

### Geometric parameters (Å, °)

**Table:**

C1—O1A	1.394 (2)	C7—H7A	0.97 (2)
C1—O14	1.441 (2)	C7A—C8	1.561 (3)
C1—O2	1.447 (2)	C8—C9A	1.545 (3)
C1—C15	1.516 (3)	C8—C15	1.545 (3)
O1A—C1B	1.471 (3)	C8—C8A	1.549 (3)
C1B—C1C	1.506 (3)	C8A—H81	0.9600
C1B—H1B1	1.01 (2)	C8A—H82	0.9600
C1B—H1B2	1.03 (2)	C8A—H83	0.9600
C1C—H1C1	0.9600	C9A—C9	1.396 (3)
C1C—H1C2	0.9600	C9A—C13	1.406 (3)
C1C—H1C3	0.9600	C9—C10	1.404 (3)
O2—C3	1.402 (2)	C9—H9A	1.02 (2)
C3—C7A	1.408 (3)	C10—C11	1.412 (3)
C3—C4	1.417 (3)	C10—H10A	1.01 (2)
C4—C5	1.401 (3)	C11—C12	1.404 (3)
C4—H4A	1.00 (2)	C11—C11A	1.521 (3)
C5—C6	1.412 (3)	C11A—H111	0.9600
C5—C5A	1.537 (3)	C11A—H112	0.9600
C5A—H51	0.9600	C11A—H113	0.9600
C5A—H52	0.9600	C12—C13	1.404 (3)
C5A—H53	0.9600	C12—H12A	0.97 (2)
C6—C7	1.416 (3)	C13—O14	1.394 (2)
C6—H6A	1.01 (2)	C15—H15A	1.03 (2)
C7—C7A	1.410 (3)	C15—H15B	1.01 (2)
			
O1A—C1—O14	106.32 (16)	C9A—C8—C15	105.53 (16)
O1A—C1—O2	106.81 (16)	C9A—C8—C8A	113.52 (16)
O14—C1—O2	107.79 (15)	C15—C8—C8A	109.48 (16)
O1A—C1—C15	110.49 (16)	C9A—C8—C7A	108.30 (15)
O14—C1—C15	112.89 (17)	C15—C8—C7A	107.35 (16)
O2—C1—C15	112.18 (17)	C8A—C8—C7A	112.28 (16)
C1—O1A—C1B	115.89 (15)	C8—C8A—H81	109.5
O1A—C1B—C1C	107.24 (18)	C8—C8A—H82	109.5
O1A—C1B—H1B1	109.9 (12)	H81—C8A—H82	109.5
C1C—C1B—H1B1	111.0 (13)	C8—C8A—H83	109.5
O1A—C1B—H1B2	109.0 (12)	H81—C8A—H83	109.5
C1C—C1B—H1B2	111.9 (12)	H82—C8A—H83	109.5
H1B1—C1B—H1B2	107.7 (17)	C9—C9A—C13	117.17 (19)
C1B—C1C—H1C1	109.5	C9—C9A—C8	123.89 (19)
C1B—C1C—H1C2	109.5	C13—C9A—C8	118.92 (17)
H1C1—C1C—H1C2	109.5	C9A—C9—C10	120.9 (2)
C1B—C1C—H1C3	109.5	C9A—C9—H9A	119.4 (11)
H1C1—C1C—H1C3	109.5	C10—C9—H9A	119.7 (11)
H1C2—C1C—H1C3	109.5	C9—C10—C11	121.6 (2)
C3—O2—C1	117.55 (15)	C9—C10—H10A	120.4 (12)
O2—C3—C7A	122.45 (18)	C11—C10—H10A	118.0 (12)
O2—C3—C4	115.67 (17)	C12—C11—C10	117.93 (19)
C7A—C3—C4	121.88 (19)	C12—C11—C11A	120.07 (19)
C5—C4—C3	121.08 (19)	C10—C11—C11A	121.97 (19)
C5—C4—H4A	119.3 (12)	C11—C11A—H111	109.5
C3—C4—H4A	119.6 (12)	C11—C11A—H112	109.5
C4—C5—C6	117.72 (19)	H111—C11A—H112	109.5
C4—C5—C5A	121.32 (18)	C11—C11A—H113	109.5
C6—C5—C5A	120.93 (18)	H111—C11A—H113	109.5
C5—C5A—H51	109.5	H112—C11A—H113	109.5
C5—C5A—H52	109.5	C13—C12—C11	119.6 (2)
H51—C5A—H52	109.5	C13—C12—H12A	120.0 (14)
C5—C5A—H53	109.5	C11—C12—H12A	120.4 (14)
H51—C5A—H53	109.5	O14—C13—C12	114.50 (18)
H52—C5A—H53	109.5	O14—C13—C9A	122.64 (18)
C5—C6—C7	120.8 (2)	C12—C13—C9A	122.85 (19)
C5—C6—H6A	120.4 (11)	C13—O14—C1	119.16 (15)
C7—C6—H6A	118.8 (11)	C1—C15—C8	108.64 (17)
C7A—C7—C6	121.9 (2)	C1—C15—H15A	110.6 (12)
C7A—C7—H7A	119.4 (12)	C8—C15—H15A	107.0 (12)
C6—C7—H7A	118.7 (12)	C1—C15—H15B	110.1 (12)
C3—C7A—C7	116.55 (19)	C8—C15—H15B	110.6 (12)
C3—C7A—C8	120.42 (18)	H15A—C15—H15B	109.9 (17)
C7—C7A—C8	122.99 (17)		
			
O14—C1—O1A—C1B	52.0 (2)	C8A—C8—C9A—C9	27.8 (3)
O2—C1—O1A—C1B	−62.9 (2)	C7A—C8—C9A—C9	−97.6 (2)
C15—C1—O1A—C1B	174.86 (18)	C15—C8—C9A—C13	−33.8 (2)
C1—O1A—C1B—C1C	177.22 (17)	C8A—C8—C9A—C13	−153.74 (18)
O1A—C1—O2—C3	−159.04 (15)	C7A—C8—C9A—C13	80.8 (2)
O14—C1—O2—C3	87.06 (19)	C13—C9A—C9—C10	−0.3 (3)
C15—C1—O2—C3	−37.8 (2)	C8—C9A—C9—C10	178.18 (18)
C1—O2—C3—C7A	5.5 (3)	C9A—C9—C10—C11	0.2 (3)
C1—O2—C3—C4	−174.66 (17)	C9—C10—C11—C12	0.4 (3)
O2—C3—C4—C5	178.45 (17)	C9—C10—C11—C11A	−177.78 (19)
C7A—C3—C4—C5	−1.7 (3)	C10—C11—C12—C13	−0.8 (3)
C3—C4—C5—C6	−0.6 (3)	C11A—C11—C12—C13	177.37 (19)
C3—C4—C5—C5A	177.54 (18)	C11—C12—C13—O14	−178.21 (17)
C4—C5—C6—C7	2.0 (3)	C11—C12—C13—C9A	0.7 (3)
C5A—C5—C6—C7	−176.14 (19)	C9—C9A—C13—O14	178.71 (18)
C5—C6—C7—C7A	−1.2 (3)	C8—C9A—C13—O14	0.1 (3)
O2—C3—C7A—C7	−177.66 (17)	C9—C9A—C13—C12	−0.1 (3)
C4—C3—C7A—C7	2.5 (3)	C8—C9A—C13—C12	−178.73 (18)
O2—C3—C7A—C8	0.3 (3)	C12—C13—O14—C1	−174.43 (17)
C4—C3—C7A—C8	−179.47 (18)	C9A—C13—O14—C1	6.6 (3)
C6—C7—C7A—C3	−1.1 (3)	O1A—C1—O14—C13	144.54 (16)
C6—C7—C7A—C8	−179.04 (18)	O2—C1—O14—C13	−101.23 (18)
C3—C7A—C8—C9A	−88.6 (2)	C15—C1—O14—C13	23.2 (2)
C7—C7A—C8—C9A	89.3 (2)	O1A—C1—C15—C8	−177.26 (16)
C3—C7A—C8—C15	24.9 (2)	O14—C1—C15—C8	−58.4 (2)
C7—C7A—C8—C15	−157.21 (19)	O2—C1—C15—C8	63.7 (2)
C3—C7A—C8—C8A	145.29 (18)	C9A—C8—C15—C1	61.0 (2)
C7—C7A—C8—C8A	−36.8 (3)	C8A—C8—C15—C1	−176.52 (17)
C15—C8—C9A—C9	147.7 (2)	C7A—C8—C15—C1	−54.4 (2)
